# From Decoction to Microencapsulation: *Opuntia ficus-indica* Flowers as a Functional Polyphenol Source for Dietary Supplements

**DOI:** 10.3390/foods15173142

**Published:** 2026-09-04

**Authors:** Carla Buzzanca, Francesco Paolo Bonomo, Angela D’Amico, Rosa Maria Dina, Vita Di Stefano, Mariano Licciardi

**Affiliations:** 1 Department of Biological, Chemical and Pharmaceutical Sciences and Technologies (STEBICEF), University of Palermo, Via Archirafi 30, 90123 Palermo, Italy; carla.buzzanca@unipa.it (C.B.); angela.damico02@unipa.it (A.D.); rosamaria.dina@unipa.it (R.M.D.); mariano.licciardi@unipa.it (M.L.); 2 Advanced Technologies Network Center (ATeN Center), Università degli Studi di Palermo, 90100 Palermo, Italy; francescopaolo.bonomo@unipa.it

**Keywords:** spray-drying, bioaccessibility, polyphenols, quercetin, microparticles, *Opuntia ficus-indica*

## Abstract

*Opuntia ficus-indica* flowers represent a valuable source of phenolic compounds with promising antioxidant properties, although their application is limited by the poor stability and bioavailability of these bioactives. This study aimed to identify the most effective extraction method for maximizing polyphenol recovery and to develop a spray-dried microparticulate delivery system capable of improving the stability and release of the freeze-dried extract. Different extraction techniques were compared by determining total phenolic content (TPC), antioxidant activity and individual phenolic compounds by HPLC-UV. Among the investigated methods, decoction proved to be the most efficient, exhibiting the highest TPC (27.59 mg GAE/g), antioxidant capacity (DPPH: 0.53 mmol TEAC/100 g d.e.; ABTS: 0.79 mmol TEAC/100 g d.e.; FRAP: 43.18 mg Fe^2+^/100 g d.e.) and the greatest overall recovery of polyphenols. HPLC analysis identified quercetin as the predominant flavonoid (57.07 mg/g), followed by kaempferol rhamnoside and quercetin-3-rhamnoside. The selected extract was successfully encapsulated by spray-drying, producing spherical microparticles (MPs) with a process yield of 64%. SEM, DSC and FT-IR analyses confirmed suitable morphology and enhanced thermal stability. In vitro dissolution and ex vivo permeation studies demonstrated a faster initial release and enhanced early quercetin permeation of MPs across porcine colon mucosa compared with the freeze-dried extract. These findings highlight spray-dried microparticles as a promising strategy for improving stability, bioaccessibility and nutraceutical potential of *O. ficus-indica* flower polyphenols.

## 1. Introduction

*Opuntia ficus-indica* (L.) Mill., commonly known as prickly pear cactus, is a xerophytic plant distributed in arid and semi-arid regions, particularly in the Mediterranean basin, Central and South America and Africa [1,2]. Historically valued across traditional medicine, agrarian practices and human nutrition, this botanical species has gained increasing scientific focus owing to its diverse bioactive composition and therapeutic potential [3,4]. Different anatomical parts of the species, such as the cladodes, fruits, seeds, and flowers, are rich sources of bioactive constituents, including polyphenols, flavonoids, betalains, vitamins, and essential minerals, which together exert antioxidant, anti-inflammatory and metabolic-modulating properties [5,6].

While most studies have focused on fruits and cladodes, the flowers of *Opuntia ficus-indica* remain relatively underexplored despite their potential as a valuable source of phenolic compounds, particularly flavonoids such as quercetin derivatives. In traditional practices, prickly pear flowers are often consumed as herbal infusions or decoctions, suggesting that aqueous extraction methods may be effective in recovering their bioactive constituents [7]. *Opuntia ficus-indica* flowers have long been used in folk medicine in Mediterranean countries, Mexico and North Africa for the management of several health conditions. Herbal infusions, beverages and decoctions prepared from the dried flowers have traditionally been consumed as diuretic, anti-inflammatory and digestive remedies, and have also been employed to relieve urinary tract disorders, kidney stones and mild gastrointestinal complaints [8]. In Sicilian and Southern Italian folk medicine, flower decoctions have been commonly used to promote diuresis and alleviate symptoms associated with prostatic hyperplasia and lower urinary tract dysfunction [9,10]. Although several biological activities have been experimentally confirmed, further investigations are still required to fully validate the traditional therapeutic uses of *O. ficus-indica* flower preparations [11,12]. However, the efficiency of different extraction techniques and their impact on the chemical composition of the resulting extracts require further investigation. A major challenge associated with plant-derived bioactive compounds is their limited stability and bioavailability, often due to sensitivity to environmental conditions such as light, oxygen, temperature and pH [13,14]. The growing consumer demand for functional and plant-based products has stimulated the development of innovative formulations incorporating natural extracts in different sectors [15,16]. Freeze-dried extracts, thanks to their low moisture content, long shelf life and ease of handling, are also easily incorporated into functional foods, supplements, beverages and cosmetics. Their concentrated nature provides high amounts of bioactives in small doses, maximizing product functionality and formulation flexibility [17], contributing to the valorization of agricultural by-products and underutilized plant materials, supporting the principles of sustainability and circular economy [18]. To protect these sensitive compounds and improve their stability and biological delivery, microencapsulation is widely employed. Among the available techniques, spray-drying is preferred for its versatility, scalability and suitability for processing heat-sensitive extracts into stable microparticles [19]. Microencapsulation based on biocompatible and food-grade materials has gained increasing attention as an effective strategy to overcome the intrinsic limitations of many bioactive compounds. This approach enhances dissolution and gastrointestinal stability, reduces degradation and enables controlled release profiles. When applied to food systems, these microcapsules demonstrated the ability to delay spoilage, preserve sensory attributes and prolong shelf life [20]. Continued research and technological innovation are expected to further optimize extraction and stabilization processes, improving the bioavailability, efficacy and commercial applicability of these ingredients. A notable research gap in the current literature is the lack of systematic comparative evaluations regarding extraction-method selection for Opuntia flower bioactives. While traditional aqueous decoction is an eco-friendly and cost-effective extraction process, there is limited understanding of how this traditional green extraction compares in yield, composition and technological applicability when transformed into a solid delivery system. Furthermore, to minimize the susceptibility of sensitive plant polyphenols to oxidative degradation during aqueous thermal extraction and subsequent processing, the application of nitrogen pretreatment can represent a key technological safeguard, effectively removing dissolved oxygen from the medium and thus creating an anaerobic atmosphere that prevents auto-oxidation and degradation of thermolabile polyphenolic bioactive compounds.

The novelty of this study lies in the development and functional validation of a tailored spray-dried delivery system for *Opuntia ficus-indica* flower decoction. This approach converts an unstable liquid extract into a stable, highly dispersible microparticulate powder. To preserve the thermosensitive bioactive profile of crude aqueous decoctions, freeze-drying serves as a critical preliminary stabilization step, removing moisture under low temperature and pressure to yield a concentrated, solid-state extract powder. Subsequently, microencapsulation techniques are applied to incorporate this freeze-dried phytocomplex into protective polymeric matrices (such as maltodextrin, mannitol and gum arabic). This sequential approach mitigates hygroscopicity, protects polyphenols from environmental degradation and enables controlled release behavior suitable for functional food applications. The physicochemical and functional properties of the obtained systems were compared by evaluating total phenolic content (TPC), antioxidant activity (DPPH, ABTS, and FRAP assays) and the phenolic profile using HPLC-UV. The selected extract was subsequently encapsulated by spray-drying to produce microparticles, which were characterized by scanning electron microscopy (SEM), differential scanning calorimetry (DSC) and Fourier-transform infrared spectroscopy (FT-IR). Finally, the performance of the microparticles was assessed through in vitro dissolution and ex vivo permeation studies across porcine colon mucosa.

## 2. Materials and Methods

### 2.1. Materials

Dulbecco’s Phosphate Buffer Saline (DPBS), ethanol, methanol, formic acid and glacial acetic acid (GAA) were purchased from Sigma Aldrich, Merck, Darmstadt, Germany. Quercetin (≥95% purity) was purchased from A.C.E.F., Fiorenzuola d’Arda, PC, Italy; NaOH, ACN and HCl were purchased from VWR, Milano, Italy; maltodextrin, mannitol and gum arabic were purchased from Galeno s.r.l. Carmignano, PO, Italy; Fresh *Opuntia ficus-indica* (L.) Mill flowers were harvested during the peak blooming season in May 2025, from local plantations in Misilmeri, Sicily, Italy. Following collection, the botanical material was authenticated and thoroughly washed with distilled water to remove surface impurities. The flowers were then air-dried in the shade at room temperature (25 ± 2 °C) until a constant weight was reached, ensuring the preservation of the thermolabile bioactives in airtight glass containers and protected from light until further use.

### 2.2. Methods

#### 2.2.1. Extraction Methods

Four different aqueous extraction techniques were evaluated: decoction, infusion, cold extraction (maceration) and extraction with liquid nitrogen pre-treatment (Table 1). For all extraction methods, the same amount of plant material was used (3 g, corresponding to approximately 8 dry flowers in 200 mL water). All extracts were finally filtered through paper filters. The filtered extracts were subsequently freeze-dried in order to obtain a freeze-dried extract (d.e.). In liquid nitrogen pre-treatment, the collected aqueous extract was first pre-frozen at −80 °C for 10 min to ensure complete ice crystal formation. Lyophilization was subsequently conducted using a laboratory freeze-dryer (Freeze Dryer Alpha 1–2 LDplus, Martin Christ Gefriertrocknungsanlagen, Osterode am Harz, Germany) operated at a condenser temperature of −55 °C under a vacuum pressure below 0.05 mbar for 48 h. The resulting freeze-dried extract powder was collected and stored in airtight, light-protected containers at −20 °C until further analysis and microencapsulation.

#### 2.2.2. Preparation of Microparticle Formulation

For the preparation of microparticles (MPs), a formulation based on *Opuntia ficus-indica* flowers was developed with a Mini Spray-Dryer B-290 (Buchi, Essen, Germany). To prepare the feed dispersion, the freeze-dried extract (1.5 g) was blended with a carrier combination consisting of maltodextrin (900 mg), mannitol (900 mg) and gum arabic (300 mg) and dissolved in ultra-pure water under magnetic stirring for 30 min and homogenized with an T 50 digital ULTRA-TURRAX^®^, Staufen, Germany at 24,000 rpm for 5 min to reach a final volume of 25 mL, corresponding to a total solids concentration of 14.4% *w*/*v*. The specific extract-to-carrier ratio 1:1.4 *w*/*w* was selected based on preliminary optimization trials. This ratio was identified as optimal to achieve powder recovery yield while preventing excessive viscosity, nozzle clogging and chamber wall-stickiness during the atomization process. Spray-drying was carried out under the following conditions: inlet temperature 130 °C, outlet temperature 70–73 °C, aspiration rate 100%, feed flow 10% (4.5 mL min^−1^), atomizer nozzle diameter 0.7 mm and compressed air as the drying gas.

The process yield (%Y) was calculated according to the following equation (Equation (1)):

(1) $$\%Y=\frac{Mass\;of\;microparticles\;obtained}{Mass\;of\;excipients\;and\;API\;in\;the\;formulation}\times 100$$

#### 2.2.3. In Vitro Dissolution Studies

An amount (200 mg) of microparticle delivery systems (MPs) and freeze-dried extract (200 mg) were each placed into a paper filter bag (cut-off 0.45 μm) in separate beakers containing 500 mL of 1 M HCl (pH 1). Sample quantities were selected based on the proportions of the functional unit dose of quercetin. All tests were conducted under strict sink conditions to prevent mass-dependent saturation effects and ensure that the observed dissolution kinetics reflected the intrinsic release mechanisms of the matrix. Continuous agitation at 100 rpm was maintained using magnetic stir bars in a thermostated water bath at 37 °C. To accommodate the small dimensions of the microparticles and avoid the limitations of traditional dissolution baskets, a filter bag technique was employed. This strategy prevents sample loss while guaranteeing full and homogeneous exposure to the dissolution medium, facilitating consistent and reproducible release behavior [21].

Aliquots of 2.0 mL were withdrawn at defined time points (0 min, 30 min, 1 h, and 2 h) and immediately replaced with an equivalent volume of pre-warmed 1 M HCl to maintain sink conditions and a constant fluid volume. Following 2 h of gastric exposure, the sample-containing filter bags were transferred to 500 mL beakers containing phosphate-buffered saline (PBS, pH 6.8) to simulate the intestinal environment. Sampling continued at scheduled time points (2.5, 3, 4, 5, 6, 7, and 8 h), replacing 2.0 mL of medium with fresh PBS at each interval. Samples were passed through 0.22 μm regenerated cellulose (RC) syringe filters, collected in 12 mL Falcon tubes, frozen at −80 °C for 24 h, and lyophilized. The dried residues were reconstituted in 2.0 mL ethanol, sonicated, refiltered (RC 0.22 μm), and analyzed for quercetin concentration at 374 nm using a Jasco V-760 UV–Vis spectrophotometer (1 mL cuvette, 10 mm path length). All testing was conducted in triplicate [22].

#### 2.2.4. Ex Vivo Permeation Studies

Ex vivo permeation studies were performed using vertical Franz diffusion cells mounted with porcine colon mucosa, incubated in an orbital shaker at 37 °C and 80 rpm. The receptor chamber was filled with 5 mL of phosphate buffer, whereas the donor chamber received 1 mL of 1 M HCl containing either microparticles (200 mg) or the crude freeze-dried extract (200 mg). Following a 2-h gastric phase simulation, the donor pH was adjusted to 6.8 via the addition of 1 M NaOH and maintained for the remaining 22 h. Samples (200 µL) were collected at predetermined intervals (0.5, 1, 2, 3, 4, 6, 8, and 24 h) and replaced with an equal volume of fresh receiver fluid. High-performance liquid chromatography (HPLC) was carried out using an Agilent 1260 Infinity setup equipped with a Quaternary Pump VL G1311C and MWD G7165A detector, driven by OpenLAB CDS ChemStation Workstation software (LTS 1.11). Chromatographic separation was achieved on a Phenomenex Luna C18 column (250 × 4.6 mm, 5 µm) using a mobile phase of 40% ACN and 60% water containing 2% glacial acetic acid (GAA) (detailed parameters in Table 2). Quercetin was detected at its maximum absorption wavelength in positive polarity mode and quantified via an external calibration curve prepared across various concentrations in ACN. Results represent the mean ± S.D. of three independent determinations.

#### 2.2.5. Phenolic Profiling by HPLC-UV

MPs and freeze-dried extract samples were analyzed to determine main phenolic compounds. The HPLC-UV analysis was performed on Thermo Scientific Vanquish system, equipped with a quaternary pump (Vanquish™ Quaternary PumpC, VC-P20-A01), an automatic injector (Vanquish™ Split Sampler C, VC-A13-A-02) and a UV detector (Vanquish™ Variable Wavelength Detector, VC-D40-A-01) (Thermo Scientific, Waltham, MA, USA). The chromatographic separation was achieved using a ReproSil Saphir 100 C18 (200 mm × 4.0 mm, 5 μm stationary phase, Dr. Maisch, Ammerbuch-Entringen, Germany), equipped with pre-column. Gradient separation using 0.1% formic acid in water (*v*/*v*) (solvent A) and methanol (solvent B) as mobile phases was as follows: 0–3 min, linear gradient from 5 to 25% B; 3–6 min, at 25% B; 6–9 min, from 25 to 37% B; 9–13 min, at 37% B; 13–18 min, from 37 to 54% B; 18–22 min, at 54% B; 22–26 min, from 54 to 95% B; 26–29 min, at 95% B; 29–29.15 min, back to initial conditions at 5% B; and from 29.15 to 36 min, at 5% B. The initial conditions were maintained for 3 min to equilibrate the column. The column was maintained at 25 °C and the flow rate was 1 mL/min. The volume of injection was 25 μL. After a series of tests of different literature-based wavelengths, values of 280 nm and 374 nm were determined optimal for obtaining signals with maximum intensity. For quantitative analysis, calibration curves were constructed for the identified compounds using standards with concentrations ranging from 10 μg/mL to 50 μg/mL. Systematic control data collection and analysis were performed using the Thermo Scientific™ Chromeleon™ 7.2 Chromatography Data System software. Analyses were carried out in triplicate, and the results were expressed as mg of phenolic compound per g of *Opuntia ficus-indica* flowers.

#### 2.2.6. Evaluation of Total Phenolic Content (TPC)

Total phenolic content (TPC) was determined using the optimized Folin–Ciocâlteu colorimetric method [23]. One g of MPs and freeze-dried extract were mixed with 12 mL of methanol/water (80:20 *v*/*v*), sonicated for 45 min, and filtered (PTFE 0.45 μm). The filtrate aliquot (0.120 mL) was incubated with 120 μL of Na_2_CO_3_ solution and 625 μL of Folin–Ciocalteu reagent (1:5) in the dark at 25 °C for 60 min. Absorbance was evaluated at λ = 765 nm (Varian Cary 50 spectrophotometer, Agilent Technologies, Milano, Italy); methanol was used as a blank, and the calibration curve was constructed with gallic acid (GA) (0.001 to 0.25 mg/mL). TPC was expressed as mg GA equivalents per g (mg GAE × g^−1^) of sample.

#### 2.2.7. Antioxidant Properties Evaluation

The determination of antioxidant activity was carried out following previously described procedures with slight modifications [24]. A total of 0.500 g of each sample was solubilized in 8 mL of an 80:20 MeOH/H_2_O solution and vortexed. The mixtures were then sonicated in an ultrasonic bath for 1 h. The supernatants were filtered through Whatman 0.45 μm PTFE filters (Merck, Darmstadt, Germany). The resulting filtrates were used for the determination of antiradical activity via DPPH, ABTS and FRAP assays. Antioxidant activity was assessed by measuring the decrease in the solution’s color, which is proportional to the amount of antioxidant present in the sample.

##### DPPH Assay

Radical scavenging activity was evaluated using the DPPH assay [2,2-diphenyl-1- 3 picrylhydrazyl], 100 μL aliquots of each sample were mixed with 3 mL of a 60 μM DPPH solution. For the MPs sample, 1 g of sample was extracted with 8 mL of methanol, sonicated, and filtered. Then, 100 μL of the filtrate was mixed with 3 mL of DPPH (60 μM). The mixtures were kept in the dark for 30 min at 25 °C. Antiradical activity was evaluated by measuring absorbance at 517 nm using a UV–Vis spectrophotometer (UV-1600 PC, VWR, Milan, Italy), with methanol used as blank. Trolox was used as the reference antioxidant standard to generate a linear calibration curve over a concentration range of 5–50 μM. The regression equation obtained was y = −0.0074x + 0.6742 (R^2^ = 0.985), where y corresponds to the absorbance measured at λ = 517 nm and x denotes the Trolox concentration (μM). The radical scavenging capacity was calculated from the calibration curve and expressed as mg Trolox Equivalents per gram of dry sample (mg TEAC/g).

##### FRAP Assay

The FRAP (Ferric Reducing Antioxidant Power) assay is based on the reduction in ferric ions (Fe^3+^) to ferrous ions (Fe^2+^), which leads to a measurable color change. Briefly, the FRAP reagent was prepared by mixing 300 mM acetate buffer (pH 3.6), 10 mM TPTZ (2,4,6-tripyridyl-s-triazine) dissolved in 40 mM HCl, and 20 mM FeCl_3_ in a 10:1:1 (*v*/*v*/*v*) ratio. Then, 100 μL of each extract was mixed with 3 mL of freshly prepared FRAP reagent, incubated at 37 °C, and absorbance was measured at 593 nm after 3 min, using the FRAP working solution as a blank. FeSO_4_ was used to generate a standard calibration curve (50–2000 μg/mL). The results were expressed as mg Fe^2+^ equivalents per 100 g of dried sample.

##### ABTS Assay

For the ABTS assay, ABTS^•+^ radical cation [2,2′-azino-bis(3-ethylbenzothiazoline-6-sulfonic acid)] was prepared following a previously described method by reacting ABTS^•+^ with potassium persulfate. The test was performed by adding 3 mL of the ABTS solution to 100 μL of each previously extracted sample (sonicated and filtered); absorbance was read at λ = 734 nm 5 min after, using methanol as blank. For both antioxidant assays, results were reported as Trolox equivalent antioxidant activity (TEAC), expressed as mmol Trolox equivalents (TEAC) per 100 g of sample using a Trolox calibration curve [2.5 μM–30 μM]. All data were performed in triplicate and reported as the mean ± standard deviation (S.D.).

#### 2.2.8. Morphological Characterization of Microparticles

The size and morphology of MPs were evaluated by scanning electron microscopy (SEM) using a Phenom ProXSEM (Thermo Fisher Scientific Inc., Waltham, MA, USA). SEM observations were carried out at an accelerating voltage of 15 kV. Samples were mounted on aluminum stubs using double-sided carbon adhesive tape and subsequently dried under vacuum (13 Pa) before analysis. All measurements were carried out at 25.0 ± 0.1 °C. The average diameter (d) ± standard deviation (S.D.) (mean ± S.D., *n* = 3) of the microparticles was determined from the mean value of 100 measurements using ImageJ (Madison, WI, USA, version 1.46 v).

Fourier-Transform Infrared spectroscopy (FT-IR) was carried out on samples prepared in KBr tablets, using a Jasco FTIR-6000. Spectra result from 100 scans in the wavenumber range 4000–400 cm^−1^, with a resolution of 1 cm^−1^.

DSC thermograms of the prepared samples were obtained using a Discovery DSC (TA instruments, New Castle, DE, USA). A total of 3–5 mg of each sample were crimped in an aluminum Tzero pan with a pinhole and were exposed to a heat–cool–heat cycle from 0 °C to 250 °C at a heating rate of 2 °C/min. All samples were first annealed at 100 °C and kept isothermal for 5 min to remove any residual moisture/solvent before the analysis. A constant flow of pure nitrogen gas with a rate of 50 mL/min was applied during the measurement (*n* = 3). The data were collected and examined with TRIOS software (TA Instruments, version 5.1.1).

#### 2.2.9. Statistical Analysis

All experiments were performed in triplicate (*n* = 3), and the results are expressed as mean ± standard deviation (S.D.). Statistical analysis was performed using GraphPad Prism 6.0 software.

## 3. Results and Discussions

The results obtained from the different extraction methods were systematically compared to evaluate their effectiveness in recovering bioactive compounds and enhancing antioxidant capacity. Conventional techniques (decoction and infusion) were assessed alongside alternative approaches, including cold extraction and the use of liquid nitrogen, to investigate their impact on extraction efficiency. The comparison was carried out by analyzing the profile of the individual phenolic compounds by HPLC/UV and by the evaluation of Total Phenolic Content (TPC) and antioxidant activity through DPPH, ABTS and FRAP assays. These parameters provide complementary information on the ability of the extracts to act as radical scavengers and reducing agents. The following section discusses the observed differences among the extraction methods, highlighting how processing conditions influence the release of phenolic compounds and, consequently, the antioxidant potential of the extracts. Particular attention is given to identifying the most effective extraction strategy to be further applied in the formulation of microparticles.

Data reported in Table 3 highlight the influence of different extraction methods on the recovery of bioactive compounds and antioxidant capacity.

Among all the tested techniques, the decoction method consistently showed the highest values across most parameters. In particular, the greatest Total Phenolic Content (TPC) (27.59 mg GAE/g d.e.) in decoction indicated a more efficient extraction of phenolic compounds. This trend is supported by the antioxidant assays: decoction exhibited the highest DPPH and ABTS value (0.53–0.79 mmol TEAC/100 g d.e., respectively), as well as the highest FRAP value (43.18 mg Fe^2+^/100 g d.e.), reflecting superior reducing power. The Total Phenolic Content (TPC) of the crude freeze-dried extract reflects the natural phytocomplex composition of traditional aqueous flower decoctions. Aqueous thermal extraction inherently co-solubilizes endogenous hydrophilic macromolecules, primarily mucilages and structural polysaccharides typical of the Opuntia genus, which constitute the bulk of the dry matter. Consequently, the microencapsulation strategy was designed to stabilize the complete, unrefined aqueous extract rather than isolated pure compounds, preserving the synergistic properties of the natural matrix within the protective ternary carrier system. Although the use of liquid nitrogen (both infusion and decoction) improved extraction efficiency compared to standard infusion, these methods do not surpass the conventional decoction. Similarly, cold extraction resulted in the lowest values for all parameters, suggesting a limited ability to solubilize phenolic compounds and antioxidant molecules. Overall, the results clearly demonstrated that decoction is the most effective extraction method for maximizing both phenolic content and antioxidant activity. These findings are consistent with previous studies reporting that hot-water decoction enhances the extraction of water-soluble phenolic compounds and antioxidant constituents from several medicinal plants [25]. Our findings regarding the total phenolic content from *Opuntia ficus-indica* flowers are in line with Benayad et al., who emphasized that the recovery of bioactive polyphenols from cactus flowers is highly influenced by the extraction technology and solvent polarity, confirming the potential of cactus flowers as a valuable source of functional polyphenols. For this reason, decoction was selected as the preferred extraction technique for the subsequent development of microparticles [26].

It should be noted that the analytical extraction of polyphenolic compounds from the microparticulate matrix was conducted using an aqueous-organic solvent system, avoiding severe acid digestion to prevent chemical cleavage or degradation of acid-labile poly–phenolic moieties and thermolabile pigments; however, the lack of forced matrix disruption may leave a small fraction of sterically trapped or non-covalently bound phenols unrecovered, potentially underestimating the absolute total phenolic content.

The quali-quantitative analysis of individual polyphenols (Table 4) also highlighted differences among the extraction methods, confirming the strong influence of processing conditions on the recovery of specific compounds.

From a compositional point of view, quercetin was the most abundant compound in all extracts, reaching its highest concentration in decoction (57.07 mg/g). Similarly, kaempferol rhamnoside showed markedly higher levels in decoction (24.10 mg/g) compared to the other methods, indicating that heat treatment enhances the extraction of flavonol glycosides. Interestingly, catechin exhibited a different trend, with higher values in liquid nitrogen-assisted methods (18.19 and 20.93 mg/g for infusion and decoction, respectively), suggesting that cell disruption induced by cryogenic treatment improves the release of flavan-3-ols. In contrast, cold extraction favored the recovery of certain phenolic acids, particularly caffeic acid (13.35 mg/g) and chlorogenic acid (7.40 mg/g), which were considerably higher than in thermal methods. This may be due to reduced thermal degradation or transformation reactions occurring at higher temperatures. Liquid nitrogen treatments generally enhanced the extraction of several compounds (e.g., vanillic acid, ferulic acid, and quercetin derivatives) but did not surpass conventional decoction in terms of total yield. This suggests that while cryogenic pre-treatment improves cell structure disruption, thermal extraction remains more effective for maximizing overall polyphenol recovery [27]. A comprehensive evaluation of the chromatographic profiles and functional assays reveals that while individual extraction methods selectively influence specific phenolic classes, such as cold maceration enhancing certain phenolic acids like caffeic and chlorogenic acids, or cryogenic pre-treatment improving flavan-3-ol extraction such as catechin, the selection of decoction as the ideal candidate for microencapsulation was governed by a clear hierarchy of quantitative, biological and technological factors. Decisive priority was assigned to the recovery of the main target bioactive flavonoids, particularly quercetin and kaempferol derivatives, which constitute the primary markers for the nutraceutical potential of prickly pear flowers. In this regard, decoction achieved the highest total phenolic yield (Σ Total = 132.47 mg/g of dry flowers) and markedly enhanced the concentration of quercetin (57.07 mg/g) and kaempferol rhamnoside (24.10 mg/g). This remarkable chemical profile directly mirrored its biological efficiency, as decoction consistently exhibited top-tier antioxidant performance across all evaluated mechanisms, displaying the highest radical scavenging capacities (DPPH and ABTS) as well as the strongest ferric reducing power (FRAP). Beyond chemical and biological superiority, practical feasibility and industrial alignment played a crucial role in finalizing this choice. Compared to liquid nitrogen pre-treatments which, despite moderate improvements, demand specialized cryogenic equipment and elevated operational costs or cold extraction requiring prolonged timeframes, hot-water decoction represents a rapid, fully aqueous, cost-effective and environmentally friendly approach that directly honors traditional usage. Therefore, the convergence of maximum bioactive flavonoid yield, superior antioxidant capacity, and seamless industrial scalability provided a robust justification for selecting the decoction extract as the core matrix for spray-drying microencapsulation. For this reason, it was selected as the most suitable extraction method for subsequent applications, including microparticles formulation. The spray-drying process successfully yielded 2.4 g of microparticles, corresponding to a process yield of 64%. This yield reflects the efficiency of the formulation and process conditions in converting the freeze-dried extract into a stable powdered system. The formulation carriers were selected due to their ability to enhance the stability of bioactive compounds, improve powder recovery and promote the formation of structured microparticles with suitable technological properties [28,29]. The conversion of the freeze-dried extract into spray-dried microparticles offers several technological and practical advantages. The microparticles were obtained as a free-flowing, stable and homogeneous powder with improved handling properties, making them more suitable for industrial processing, to be formulated into oral dosage forms such as capsules, sachets, or tablets and dosing than the freeze-dried extract. In addition, the encapsulation matrix is expected to reduce the hygroscopicity of the powder and improve its physical and chemical stability during storage by protecting quercetin from environmental factors such as moisture, oxygen, and light. These characteristics facilitate accurate dose standardization and reproducible administration, which are difficult to achieve with the freeze-dried extract because of its poor flowability, higher hygroscopicity and heterogeneous powder characteristics. Therefore, the developed microparticles represent a more suitable delivery system for the development of nutraceutical formulations, providing improved technological performance together with enhanced functionality [30,31].

These results are consistent with those reported by De Leo et al. who identified quercetin and kaempferol derivatives as the major phenolic constituents of *O. ficus-indica* flowers using HPLC-PDA-ESI-MS. In particular, the predominance of quercetin derivatives observed in the present study confirms that flavonols represent the main class of bioactive compounds in prickly pear flowers. Minor differences in the relative abundance of individual phenolics between the two studies are expected and may arise from differences in geographical origin (Sicilian ecotypes), flowering stage, environmental conditions, and, most importantly, extraction methodology and analytical approach. While De Leo et al. mainly focused on the phytochemical characterization of flower extracts, the present work demonstrates that extraction technology has a decisive impact on the recovery of specific phenolic classes. The superior performance of decoction in terms of total phenolic yield and flavonol extraction, together with its higher antioxidant activity, supports its selection as the most suitable extraction method for the subsequent preparation of spray-dried microparticles [10].

Although the overall phenolic profile observed in the present study is consistent with that reported by De Leo et al., some differences in the relative abundance of individual compounds were evident. In particular, the present HPLC-UV analysis revealed a markedly higher content of free quercetin, whereas De Leo et al. predominantly identified quercetin glycosides and other flavonol conjugates by HPLC-PDA-ESI-MS. This apparent discrepancy may be explained by several factors. First, the use of decoction as the extraction method may have promoted the partial hydrolysis of flavonoid glycosides, leading to the release of the corresponding aglycones, including quercetin. Second, differences in analytical methodology are likely to have contributed to the observed variations. HPLC-ESI-MS provides superior sensitivity and structural identification of glycosylated flavonoids, whereas HPLC-UV quantification relies on chromatographic separation and UV absorbance, which may lead to different relative distributions of structurally related compounds. In addition, environmental and agronomic factors, such as geographical origin, pedoclimatic conditions, genotype, harvesting season and flower developmental stage, are well known to influence the phenolic composition of *Opuntia ficus-indica* flowers. Therefore, the differences observed between the two studies are more likely attributable to extraction and analytical procedures, together with natural phytochemical variability, rather than to inconsistencies in the phytochemical composition of the plant material itself.

Data reported in Table 5 show the quali-quantitative analysis of individual polyphenols in freeze-dried extract from decoction and MPs, by HPLC-UV analysis.

To correctly interpret the phenolic profile before and after spray-drying, the calculation basis and matrix composition must be clearly defined. While Table 4 expresses the individual compound yield relative to the raw plant material (mg/g of dry flowers) to evaluate and compare the raw extraction efficiency and the different aqueous extraction techniques, Table 5 evaluates the retention of bioactives per unit weight of the crude freeze-dried decoction extract (mg/g d.e.) and the final microparticulate delivery system (mg/g MPs). In the microparticle formulation, 1.5 g of freeze-dried decoction extract was combined with 2.1 g of carrier excipients (maltodextrin, mannitol and gum arabic), yielding a theoretical extract loading of approximately 41.7% *w*/*w* within the solid carrier matrix. Consequently, a proportional reduction in phenolic concentration per gram of microparticles is expected, since the incorporation of the extract into the carrier matrix causes a dilution effect and may also cause limited losses of some compounds during spray-drying.

The HPLC analysis revealed that both the freeze-dried extract and the spray-dried microparticles (MPs) retained a wide spectrum of phenolic acids and flavonoids, although quantitative differences were observed as a consequence of the microencapsulation process. Overall, the freeze-dried decoction exhibited higher concentrations of several phenolic compounds, including gallic acid (57.005 ± 1.64 vs. 45.658 ± 0.76 mg/g), catechin (54.064 ± 1.58 vs. 46.278 ± 0.79 mg/g), caffeic acid (20.651 ± 0.61 vs. 15.683 ± 0.12 mg/g), vanillic acid (14.040 ± 0.43 vs. 7.745 ± 0.17 mg/g), quercetin-3-rhamnoside (77.868 ± 2.31 vs. 47.275 ± 1.01 mg/g), kaempferol rhamnoside (81.832 ± 2.45 vs. 47.173 ± 1.02 mg/g), kaempferol (12.307 ± 0.38 vs. 0.550 ± 0.02 mg/g), and apigenin (1.669 ± 0.06 vs. 0.206 ± 0.01 mg/g). Interestingly, the microencapsulation process did not uniformly decrease all phenolics. Several bioactive compounds were present at higher concentrations in the MPs than in the freeze-dried decoction. In particular, chlorogenic acid increased from 11.411 ± 0.35 to 17.297 ± 0.17 mg/g, p-coumaric acid from 10.730 ± 0.33 to 16.601 ± 0.15 mg/g, quercetin-3-glucoside from 14.259 ± 0.43 to 15.842 ± 0.13 mg/g, myricetin from 12.451 ± 0.38 to 14.687 ± 0.11 mg/g, vanillin from 4.980 ± 0.16 to 5.218 ± 0.03 mg/g, while quercetin, the predominant flavonoid, showed a remarkable increase from 83.619 ± 2.48 to 93.386 ± 1.98 mg/g. The higher concentrations of these compounds may be attributed to their greater stability during spray-drying, improved extractability after encapsulation, or the protection provided by the support matrix against thermal degradation and oxidation. Of all the compounds identified, quercetin remained the major constituent in both samples, confirming that the spray-drying process effectively preserved this biologically relevant flavonol. The increased concentration of quercetin in the microparticles is particularly noteworthy considering its well-known antioxidant, anti-inflammatory, and health-promoting properties, suggesting that microencapsulation may selectively protect this compound during the process. The encapsulation matrix effectively preserved the qualitative phytochemical profile while maintaining substantial levels of the main bioactive constituents. Furthermore, the observed increase in chlorogenic acid, p-coumaric acid, quercetin-3-glucoside, myricetin, and especially quercetin demonstrates that spray-drying can enhance the stability of some phenolic compounds rather than simply causing their degradation. This behavior can be mainly attributed to two synergistic phenomena. First, thermal exposure during the spray-drying process (inlet temperature 130 °C) can promote the partial cleavage of glycosidic bonds in conjugated flavonoid complexes, converting quercetin glycosides to their corresponding aglycone form. Second, the incorporation of hydrophilic coating materials (maltodextrin, mannitol, and gum arabic) significantly improves the wettability and redispersibility of the powder during analytical sample preparation, thus improving analytical extractability and recovery of hydrophobic bioactives compared to the highly cohesive crude lyophilized extract. Overall, although the spray-drying process effectively stabilized the primary target flavonoids, while producing a free-flowing and stable powder with improved technological properties, confirming the exact mechanisms of physical protection or potential degradation pathways of individual phenolic compounds will require further specific recovery and kinetic studies. From a practical standpoint, microparticles offer significant advantages over the freeze-dried extract. In addition to preserving most of the phenolic profile, microencapsulation improves powder handling, reduces hygroscopicity, increases oxidative and thermal stability, protects sensitive phytochemicals during storage and provides a more suitable ingredient for incorporation into functional foods, nutraceuticals, and pharmaceutical formulations. Thus, despite the expected dilution effect associated with the wall material, spray-dried MPs successfully preserved the bioactive composition of the decoction while imparting improved physicochemical stability and application potential, making them a more versatile and value-added formulation than the freeze-dried extract alone [32]. As recently demonstrated by Cegledi et al., microencapsulation via spray-drying effectively creates a protective polymer barrier that protects temperature and oxygen-sensitive polyphenols from environmental degradation. From a process perspective, spray-drying significantly improves the technological functionality of the matrix by reducing residual moisture content, improving water solubility, and transforming bulky liquid extracts into stable, free-flowing powders suitable for industrial handling and long-term storage [33].

**Figure 1:**
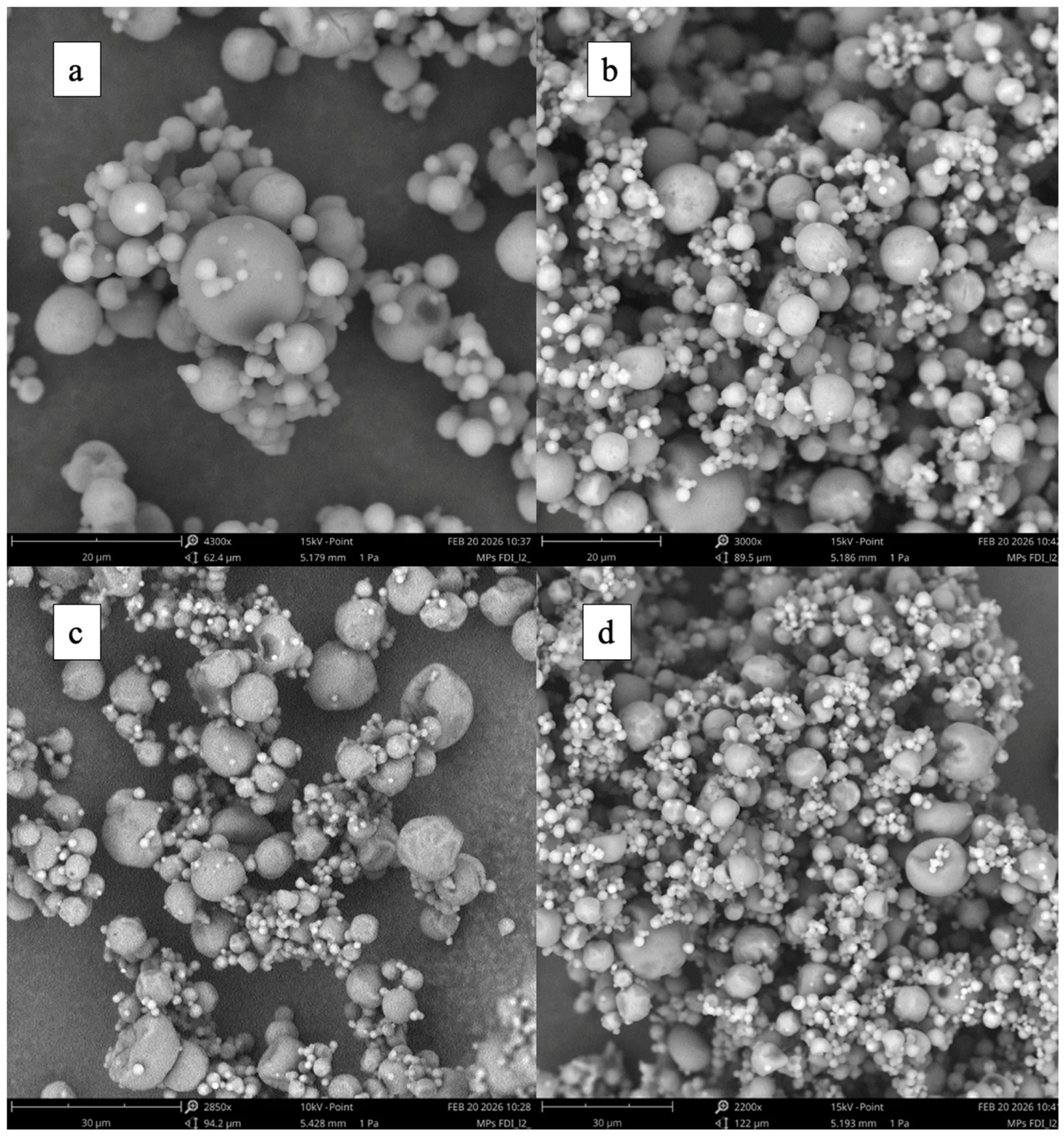
SEM image of the microparticles batches: (**a**) magnification 4300×; (**b**) magnification 3000×; (**c**) magnification 2850×; (**d**) magnification 2200×.

After their production, the microparticle delivery systems were characterized by Scanning Electron Microscopy (SEM) analysis to observe the surface characteristics and average size of the produced microparticles (Figure 1). Morphologically, the microparticles exhibited a predominantly spherical shape with characteristic surface indentations and shriveling, which is typical for spray-dried powders composed of hydrophilic polysaccharide and polyol carriers such as maltodextrin, mannitol and gum arabic due to rapid water evaporation during atomization. This morphological feature can increase the specific surface area and potentially enhance the dissolution and bioaccessibility of the encapsulated microparticles. ImageJ (Madison, WI, USA, version 1.46 v) image analysis performed on 100 individual microparticles from the SEM micrographs revealed a mean particle diameter of 4.65 ± 2.15 μm, with a particle size distribution ranging from 0.85 μm to 13.80 μm. This micrometric size range confirms the successful production of fine microparticles suitable for uniform dispersion in oral functional beverages, while the smooth and intact surfaces indicate efficient entrapment of the extract bioactives within the carrier matrix. Such a size distribution reflects the typical polydispersity of spray-dried powders, confirming the structural validity and reproducibility of the atomization process. The overall small micrometric dimensions are highly advantageous for facilitating rapid powder dispersion in aqueous environments and enhancing potential interactions with the gastrointestinal mucosa, ultimately promoting the bioavailability of the encapsulated bioactives [15]. SEM micrographs were obtained at operating voltages of 10–15 kV, with magnification factors ranging from 2200 to 4300. Scale bars are provided on each panel for quantitative dimensional assessment.

**Figure 2:**
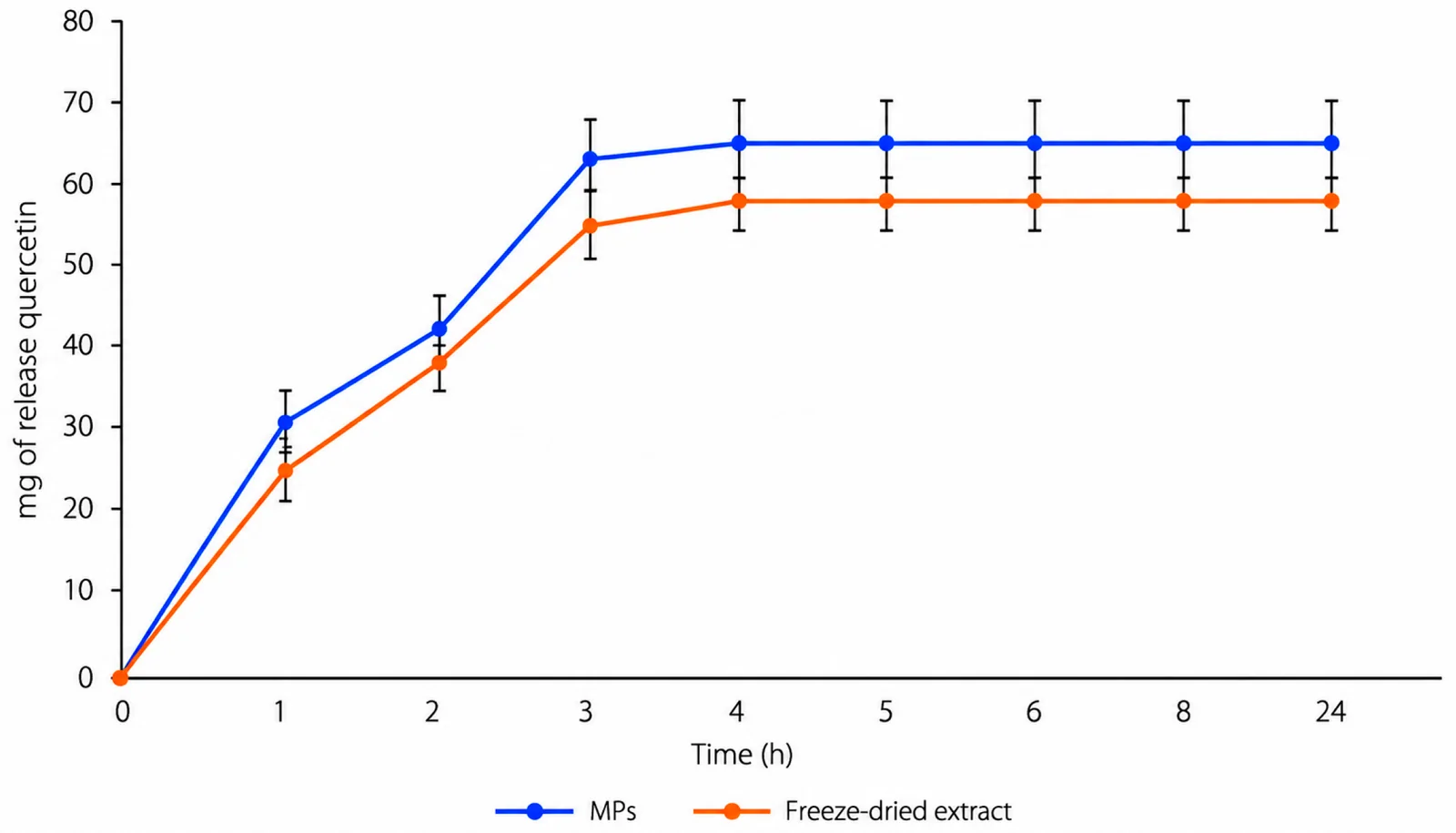
Graphs of release profiles (mg of quercetin versus time) of freeze-dried extract compared with MPs. Data are means ± S.D. *n* = 3.

An in vitro dissolution study was carried out to compare the release profile of quercetin in *Opuntia ficus-indica* flower freeze-dried extract and MPs (Figure 2). During the experiment, the release medium was maintained at pH 1–2 for the first 2 h to simulate gastric conditions and then adjusted to pH 6.8 to mimic intestinal transit.

The dissolution profile data of quercetin clearly highlight the impact of spray-drying microencapsulation on the solubilization behavior of the extract. As evidenced by the dissolution curves, the formulated microparticles exhibited a faster and higher initial release rate compared to the crude freeze-dried decoction extract. Within the early stage of the assay, the microparticulate system achieved a rapid release of quercetin (32.2 mg at t = 0.5 h), whereas the raw freeze-dried extract displayed a slower dissolution kinetic (26.6 mg at t = 0.5 h). This enhanced dissolution behavior is primarily driven by the incorporation of highly water-soluble hydrophilic wall materials, specifically maltodextrin, mannitol, and gum Arabic, which drastically improve powder wettability, decrease surface tension, and prevent particle agglomeration upon contact with the aqueous medium. In contrast, the unformulated freeze-dried extract suffers from high cohesion and poor powder wetting, which restricts solvent penetration and delays compound dissolution. Following this rapid initial dissolution phase, the microparticles maintained a consistently superior solubilization level throughout the evaluation period (64–65 mg, which remained stable up to 24 h). Overall, rather than retarding compound liberation, the microencapsulation strategy successfully serves as a fast-dissolving delivery platform, converting a cohesive plant extract into a highly dispersible powder with optimized dissolution performance suitable for nutraceutical applications. This finding suggests that microencapsulation did not substantially alter the final release extent but enhanced the early availability of the bioactive compound.

The ex vivo permeation study conducted across porcine colon mucosa, used as a model tissue, further corroborated the functional advantages of the microencapsulation strategy for oral delivery applications and evaluated the potential enhancement of permeability for microencapsulated active ingredients through the mucosa. The assay evaluated the ability of the formulated microparticles to promote polyphenol transport across the mucosal epithelial barrier relative to the crude decoction extract [34].

**Figure 3:**
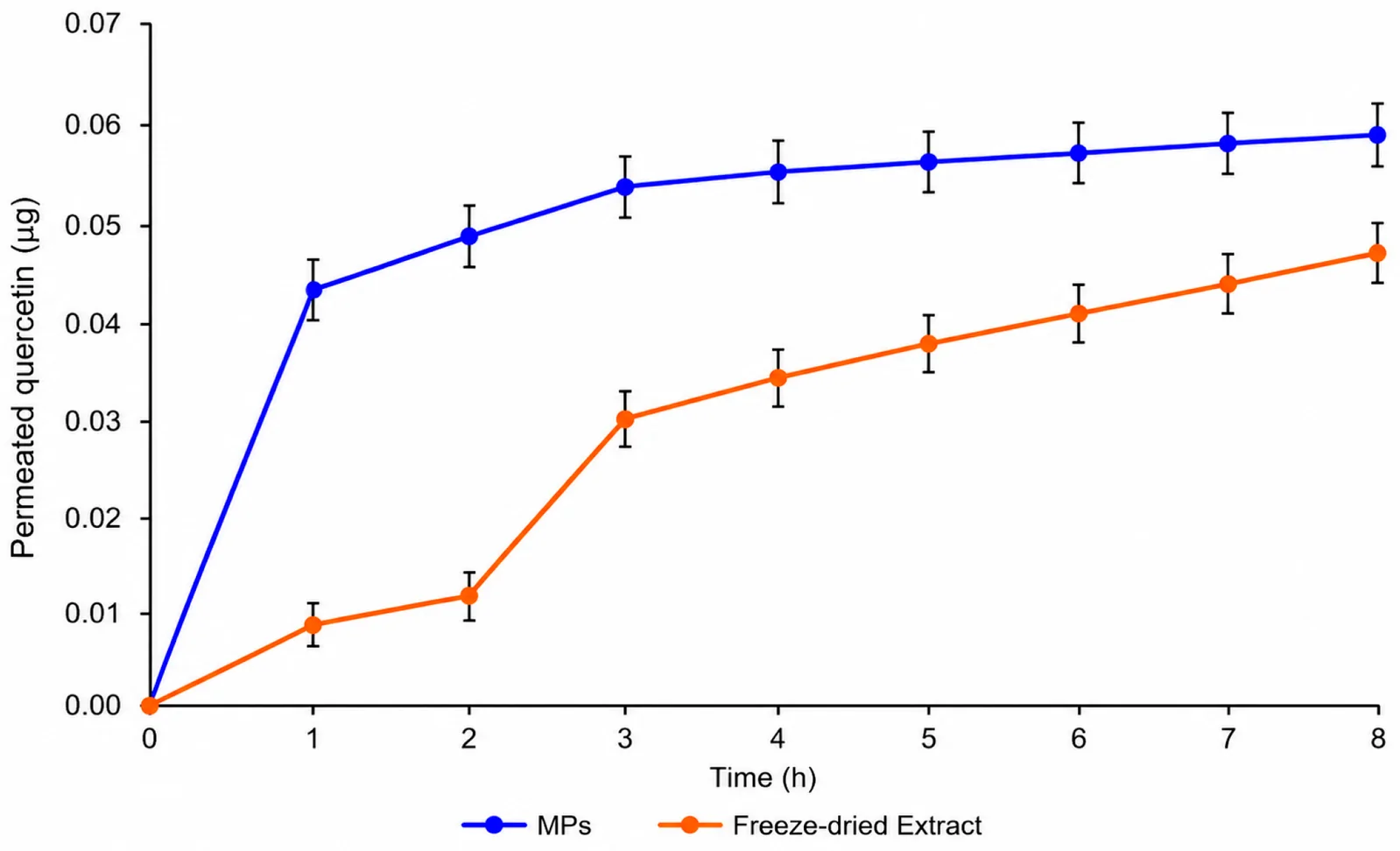
Graph of the amount (μg) of permeated quercetin as a function of time for MPs and freeze-dried extract.

As shown in Figure 3, the results demonstrated an enhanced permeation profile for the microencapsulated formulation, compared with the freeze-dried extract, which can be attributed to the synergistic action of the hydrophilic carrier matrix. A rapid increase in quercetin permeation was observed during the first hour, reaching approximately 0.044 μg, whereas the freeze-dried extract displayed a much slower and more gradual permeation. After 3 h, the MPs achieved a permeated amount of approximately 0.054 μg, approaching a plateau that was maintained until the end of the experiment, while the freeze-dried extract continued to increase progressively without reaching the same permeation level. The faster initial permeation and the higher cumulative amount of permeated quercetin obtained with the MPs indicate that the microencapsulation strategy effectively enhanced quercetin transport across the membrane. This behavior can be attributed to the permeation-enhancing effect of the microparticle formulation, which likely improved quercetin solubility, maintained a higher concentration gradient at the membrane interface, and facilitated its diffusion. The improved early permeation can also be attributed to the physicochemical characteristics of the MPs, including their reduced particle size, increased specific surface area and improved wettability, which facilitate faster dissolution. Furthermore, the polymeric matrix may promote intimate contact with the mucosal surface, increasing the residence time of the formulation and consequently enhancing the initial flux of quercetin. Conversely, the freeze-dried extract showed a delayed permeation profile, probably due to the lower apparent solubility of quercetin and the absence of a carrier system capable of promoting rapid release and transport across the biological barrier. Nevertheless, the gradual increase observed over time indicates that quercetin was progressively released and diffused through the tissue, ultimately achieving a cumulative permeated amount similar to that obtained with MPs. Consequently, the spray-drying formulation successfully overcomes the poor permeability typical of raw plant extracts, proving to be an effective oral delivery system for enhancing the intestinal absorption and nutraceutical potential of prickly pear flower polyphenols.

Overall, although both formulations delivered comparable cumulative amounts of quercetin after 8 h, the microparticles demonstrated a clear advantage in terms of permeation kinetics, enabling a faster onset of quercetin transport across the mucosa. This behaviour is particularly advantageous for oral delivery systems, where rapid drug absorption may improve bioavailability and therapeutic efficacy while reducing the residence time required to achieve effective permeation.

When evaluating the solid-state characterization (DSC, FT-IR and SEM), the baseline properties of the carrier matrix, composed of maltodextrin, mannitol, and gum arabic, must be considered. In FT-IR and DSC analyses, the predominant spectroscopic bands and thermal transitions observed in the microparticles largely reflect the structural backbone of these hydrophilic excipients. Comparing the crude freeze-dried extract directly with the final microparticles clearly demonstrates the physical state modification and successful entrapment of the active core within the carrier matrix.

The spray-drying process was utilized as a microencapsulation strategy to facilitate the incorporation and retention of the active compound within the carrier matrix (maltodextrin, mannitol and gum arabic). Comparative evaluation of formulations prepared at varying core-to-carrier ratios demonstrated that spray-drying with the selected wall materials yields a significantly higher retention of the active payload compared to unencapsulated control blends. This confirms that the rapid drying process promotes structural incorporation into the matrix rather than mere physical adhesion.

**Figure 4:**
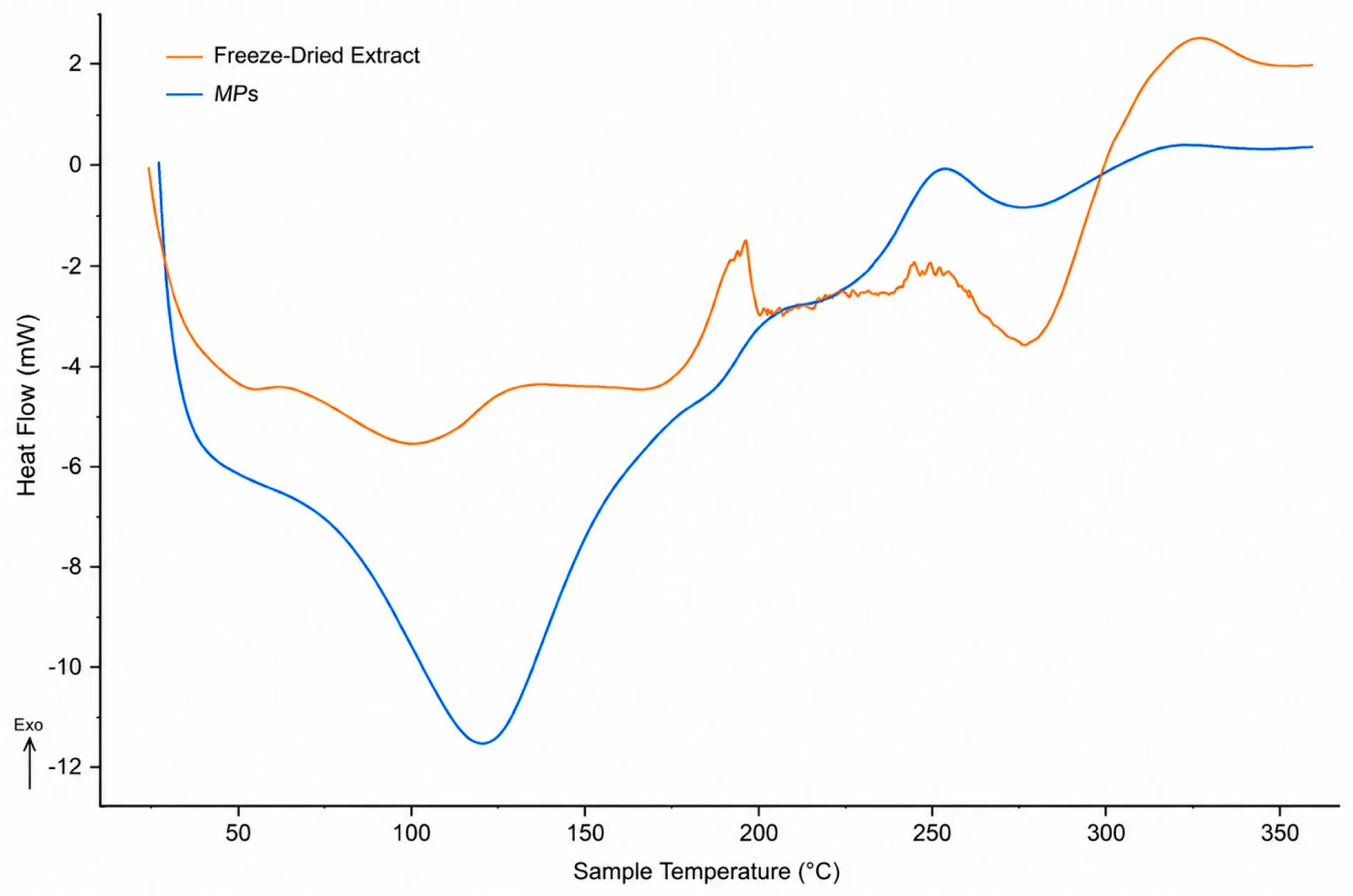
DSC thermographs of *Opuntia ficus-indica* flower freeze-dried extract and MPs.

Differential Scanning Calorimetry (DSC) was performed to characterize the thermal behavior of the freeze-dried extract and the microencapsulated powder (MPs) (Figure 4). The thermogram of the freeze-dried extract displayed an endothermic dehydration event centered at approximately 90–120 °C, which can be attributed to moisture loss and/or thermal degradation of low-molecular-weight constituents, followed by distinct exothermic thermal decomposition events at 196 °C and 277 °C. In contrast, the MPs exhibited a prominent endothermic transition centered at 120 °C (heat flow ≈ −11.5 mW), corresponding to residual moisture release and thermal transitions of the wall material matrix, alongside an exothermic transition peak at 254 °C. Notably, the characteristic decomposition events of the raw extract (196 °C and 277 °C) were no longer observable in the MPs curve. This attenuation indicates that the bioactive compounds are physically entrapped and molecularly dispersed within the polysaccharide/polyol carrier network rather than undergoing chemical reaction, which protects the core extract from direct thermal degradation.

Overall, the DSC analysis confirmed that the microencapsulation process altered the thermal transitions of the *Opuntia ficus-indica* freeze-dried extract. The absence of the characteristic decomposition peaks of the crude extract in the microparticles indicates effective physical entrapment and amorphization/molecular dispersion within the hydrophilic carrier matrix (maltodextrin, mannitol and gum arabic), which shields the active polyphenols from direct exposure to elevated processing temperatures.

**Figure 5:**
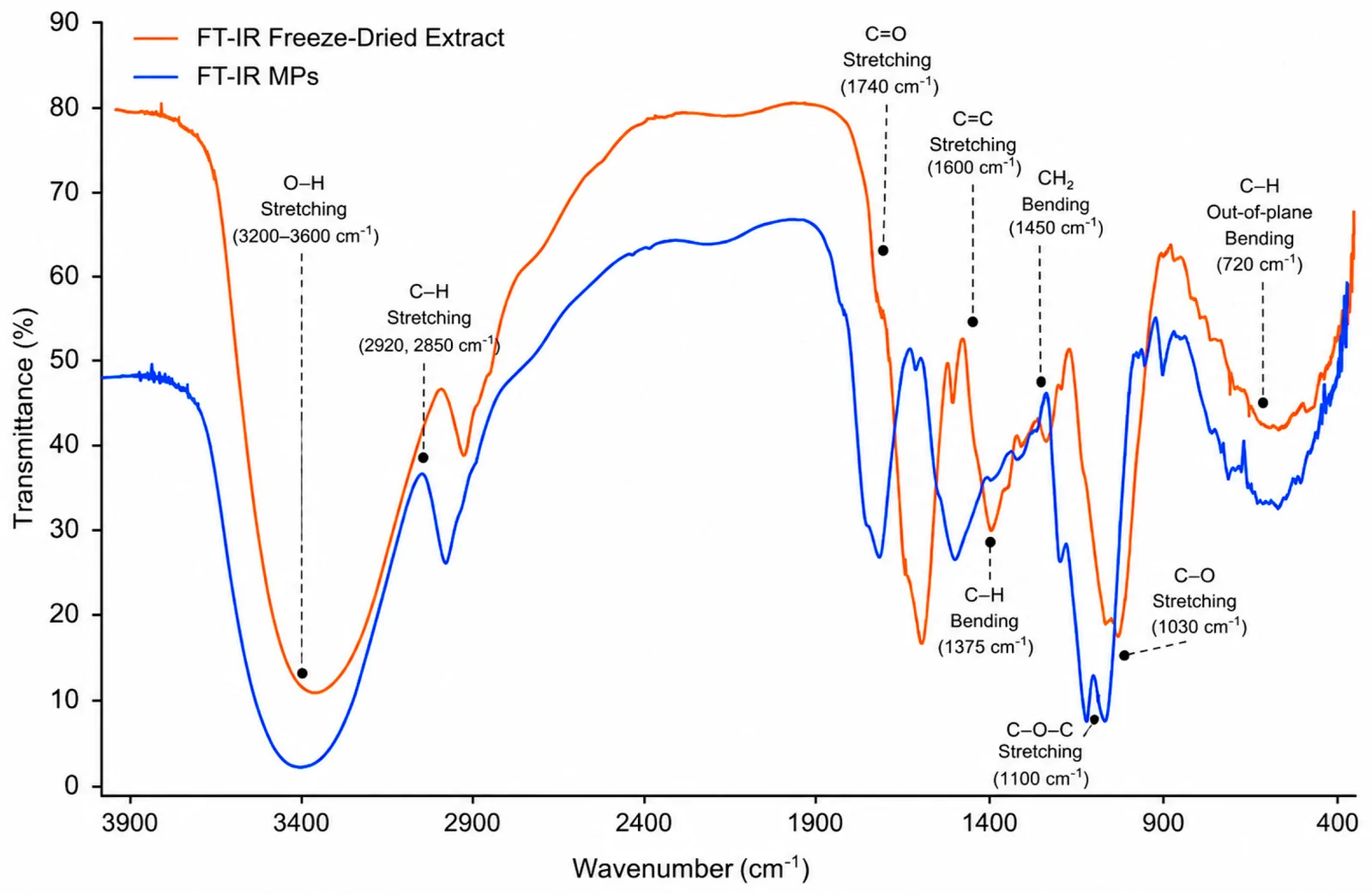
FT-IR spectra from 400 cm^−1^ to 4000 cm^−1^ of freeze-dried extract and MPs, showing characteristic peaks of the functional groups, highlighted by the labels.

FT-IR spectroscopic analysis was used to identify functional group characteristics and evaluate potential structural changes upon microencapsulation (Figure 5). The spectrum of the *Opuntia ficus-indica* freeze-dried extract exhibited characteristic absorption bands, including a broad O–H stretching vibration centered at 3400 cm^−1^ (3200–3600 cm^−1^), aliphatic C–H stretching at 2920 cm^−1^ and 2850 cm^−1^, C=O carbonyl stretching at 1740 cm^−1^, aromatic ring C=C stretching at 1600 cm^−1^, commonly found in flavonoid structures, CH_2_ bending at 1450 cm^−1^, C–H bending at 1375 cm^−1^, C–O stretching at 1030 cm^−1^ and C–H out-of-plane bending at 720 cm^−1^. The FT-IR profile confirmed the complex phytochemical composition of the freeze-dried prickly pear extract and supported the possible presence of bioactive phenolic constituents with potential antioxidant activity. In the MPs spectrum, while the major characteristic functional bands remained present, suggesting that the main chemical constituents of the freeze-dried extract, including quercetin-related flavonoids, were retained during the microparticle production process, the dominant signal shifted towards a prominent C–O–C glycosidic linkage stretching band at 1100 cm^−1^, reflecting the polysaccharide/polyol carrier composition (maltodextrin, mannitol, and gum arabic).

A qualitative comparative evaluation of the FT-IR spectra was performed by matching the observed vibrational bands of the formulated microparticles with the established spectral profiles of the individual raw carriers reported in the literature. The recorded spectra are predominantly characterized by the structural features of the hydrophilic coating matrices. Specifically, maltodextrin exhibits characteristic carbohydrate absorption bands, including a broad O–H stretching vibration, aliphatic C–H stretching and a prominent C–O–C glycosidic linkage centered stretching band. The presence of mannitol is reflected by its distinctive polyol profile, characterized by C–OH hydroxyl peaks, intense C–O stretching modes and C–H bending vibrations. Finally, gum arabic contributes to the overall spectral fingerprint through its characteristic broad O–H and alipathic C–H bands, alongside asymmetric and symmetric carboxylate stretching peaks and a C–O stretching absorption [35,36,37,38].

Overall, the FT-IR comparison demonstrated strong spectral similarity between the freeze-dried extract and the derived microparticles, confirming the efficient incorporation and the preservation of the principal functional groups after encapsulation and supporting the effectiveness of the microparticle formulation strategy for protecting *Opuntia ficus-indica* flowers’ phytochemicals. However, FT-IR analysis alone is not sufficient to unequivocally confirm quercetin, and complementary chromatographic analyses (e.g., HPLC or UHPLC-MS) are recommended for definitive identification. The FT-IR spectra of the spray-dried microparticles exhibited high spectral overlap with both the crude extract and the ternary carrier material. This similarity is attributed to shared dominant functional groups inherent to both the phenolic phytocomplex and the carbohydrate carriers (maltodextrin, mannitol, and gum arabic). Crucially, the retention of key bioactive absorption bands demonstrates that the thermal spray-drying process preserved the chemical integrity of the extract without inducing chemical degradation or covalent modification, supporting successful physical entrapment within the hydrophilic carrier matrix.

Finally, while the rapid dissolution kinetics and improved ex vivo intestinal permeation are clearly mediated by the high solubility and wetting properties of the selected wall materials, future mechanistic studies incorporating blank carrier controls will be useful to further refine the precise contribution of the matrix compared to the active extract.

## 4. Conclusions

This study demonstrated that the extraction method significantly influences the recovery of phenolic compounds and the antioxidant potential of *Opuntia ficus-indica* floral extracts. Among the techniques studied, conventional decoction proved to be the most effective, providing the highest total phenolic content, the strongest antioxidant activity, and the highest overall yield of bioactive flavonoids, particularly quercetin and kaempferol derivatives. Based on these results, the lyophilized extract obtained by decoction was successfully incorporated into freeze-dried microparticles, resulting in a stable release system with suitable morphological and physicochemical characteristics. Although microencapsulation resulted in a partial reduction in polyphenolic content, the microparticles retained substantial amounts of the main antioxidant compounds and showed thermal stability, confirming the protective effect of the encapsulating matrix. Furthermore, FT-IR analysis demonstrated that the encapsulation process preserved the main functional groups of the phytochemicals without significant chemical degradation. In vitro dissolution and ex vivo permeation studies further highlighted the advantages of microparticle formulation. Microparticles also facilitated faster initial release and improved the initial permeation rate through the biological membrane. These properties may contribute to improved bioavailability and a more rapid onset of biological activity, making the formulation particularly attractive for nutraceutical and functional food applications. Overall, spray-dried microparticles represent a promising strategy for improving the stability, handling and release of *Opuntia ficus-indica* flower polyphenols while preserving their antioxidant potential. Future studies should focus on evaluating the long-term stability of microparticles under accelerated storage conditions, the in vivo oral bioavailability and the biological efficacy of encapsulated polyphenols, in order to further validate the potential of microparticles by testing their technological performance and their incorporation into complex functional food matrices and nutraceutical supplement dosage forms. In this context, the proposed formulation aligns with the principles of the circular bioeconomy and contributes to the objectives of the United Nations 2030 Agenda, in particular Sustainable Development Goal 3 (Good health and well-being) by promoting preventive nutrition, and Sustainable Development Goal 12 (Responsible consumption and production) by encouraging the efficient use of plant-based resources.

## Figures and Tables

**Table 1:** Different aqueous extraction methods of *Opuntia ficus-indica* flowers.

Extraction Method	Procedure	Key Parameters
Decoction	Immersed in water and brought to boiling in 200 mL water	5 min; high temperature and thermal agitation enhance extraction of water-soluble compounds
Infusion (Herbal Tea)	Immersed in hot water (previously boiled) and left to infuse off heat	Moderate temperature; time-dependent extraction
Cold Extraction (Maceration)	Immersed in water at room temperature	24 h; no thermal treatment
Liquid Nitrogen Pre-treatment + Aqueous Extraction (Infusion or Decoction)	Pre-treated with liquid nitrogen, then subjected to aqueous extraction	Cryogenic disruption enhances release of intracellular compounds

**Table 2:** HPLC operating conditions.

Parameters	
**Instrument**	Agilent 1260 Infinity system
**Pump**	Vanquish™ Quaternary PumpC, VC-P20-A01
**Detector**	Vanquish™ Variable Wavelength Detector
**Software**	OpenLAB CDS ChemStation Workstation
**Injection volume**	15 μL
**Column**	Luna Phenomenex C18 (250 × 4.6 mm, 5 μm)
**Column temperature**	30 °C
**Mobile phase**	40% ACN/60% H_2_O containing 2% GAA
**Flow rate**	1 mL min^−1^
**Maximum pressure**	400 bar
**Detection wavelength**	374 nm
**Signal polarity**	Positive mode

**Table 3:** Total phenolic content (TPC) and antiradical scavenging activity (DPPH, ABTS and FRAP) of the freeze-dried extract of different extraction methods. Results indicate mean values of three determinations ± S.D. Abbreviations: TPC, total phenolic content; d.e., dry extract.

Parameter	Decoction	Infusion	Cold Extraction	Liquid Nitrogen Infusion	Liquid Nitrogen Decoction
TPC (mg GAE/g d.e.)	27.59 ± 0.82	22.28 ± 0.65	20.10 ± 0.54	23.88 ± 0.71	26.74 ± 1.58
DPPH (mmol TEAC/100 g d.e.)	0.53 ± 0.02	0.23 ± 0.01	0.13 ± 0.01	0.23 ± 0.01	0.36 ± 0.02
ABTS (mmol TEAC/100 g d.e.)	0.79 ± 0.03	0.51 ± 0.02	0.45 ± 0.02	0.68 ± 0.03	0.66 ± 0.03
FRAP (mg Fe^2+^/100 g d.e.)	43.18 ± 1.12	35.98 ± 0.94	18.98 ± 0.57	37.98 ± 1.01	38.58 ± 1.09

**Table 4:** Single phenolic compounds in different extraction methods identified by HPLC-UV (mg/g flowers). Results indicate mean values of three determinations ± S.D.

Compound	Decoction	Infusion	Cold Extraction	Liquid Nitrogen Infusion	Liquid Nitrogen Decoction
			mg/g		
Gallic acid	10.49 ± 0.31	10.90 ± 0.34	5.31 ± 0.18	10.36 ± 0.30	10.20 ± 0.29
Catechin	11.21 ± 0.35	11.37 ± 0.32	8.54 ± 0.26	18.19 ± 0.52	20.93 ± 0.60
Chlorogenic acid	3.43 ± 0.11	1.60 ± 0.06	7.40 ± 0.22	5.08 ± 0.16	3.13 ± 0.10
Caffeic acid	1.30 ± 0.05	1.51 ± 0.05	13.35 ± 0.41	6.80 ± 0.21	2.03 ± 0.07
Vanillic acid	1.84 ± 0.06	1.85 ± 0.06	1.42 ± 0.05	3.04 ± 0.11	3.64 ± 0.11
Epicatechin	0.04 ± 0.002	0.19 ± 0.008	0.06 ± 0.003	0.27 ± 0.01	0.23 ± 0.01
Syringic acid	0.11 ± 0.005	0.06 ± 0.003	0.06 ± 0.003	0.06 ± 0.003	0.02 ± 0.001
Vanillin	0.21 ± 0.01	0.74 ± 0.03	0.34 ± 0.02	0.84 ± 0.003	1.11 ± 0.04
p-Coumaric acid	1.50 ± 0.05	2.08 ± 0.07	0.65 ± 0.03	2.28 ± 0.08	1.52 ± 0.05
Ferulic acid	0.78 ± 0.03	0.33 ± 0.01	0.18 ± 0.01	1.07 ± 0.04	1.35 ± 0.05
Quercetin-3-glucoside	4.32 ± 0.14	1.96 ± 0.07	0.63 ± 0.03	4.69 ± 0.15	4.20 ± 0.13
Quercetin-3-rhamnoside	15.04 ± 0.43	7.02 ± 0.21	2.40 ± 0.08	4.29 ± 0.14	3.16 ± 0.10
Myricetin	0.68 ± 0.03	1.08 ± 0.04	n.d.	1.49 ± 0.05	3.02 ± 0.09
Kaempferol rhamnoside	24.10 ± 0.72	14.0 ± 0.42	2.20 ± 0.08	3.92 ± 0.12	7.64 ± 0.23
Kaempferol	0.20 ± 0.01	0.08 ± 0.003	n.d.	0.01 ± 0.001	0.43 ± 0.02
Quercetin	57.07 ± 1.65	35.14 ± 1.02	n.d.	32.57 ± 0.98	53.19 ± 1.56
Apigenin	0.13 ± 0.005	0.09 ± 0.004	n.d.	0.21 ± 0.008	0.18 ± 0.007
**Σ Total**	**132.47**	**89.99**	**42.52**	**95.17**	**115.98**

**Table 5:** Single phenolic compounds in freeze-dried extract from decoction (mg/g freeze-dried extract) and MPs (mg/g microparticles) identified by HPLC-UV.

Compound	Decoction Freeze-Dried Extract	MPs
	mg	
Gallic acid	57.005 ± 1.64	45.658 ± 0.76
Catechin	54.064 ± 1.58	46.278 ± 0.79
Chlorogenic acid	11.411 ± 0.35	17.297 ± 0.17
Caffeic acid	20.651 ± 0.61	15.683 ± 0.12
Vanillic acid	14.040 ± 0.43	7.745 ± 0.17
Epicatechin	0.950 ± 0.04	0.687 ± 0.02
Syringic acid	1.560 ± 0.06	0.958 ± 0.03
Vanillin	4.980 ± 0.16	5.218 ± 0.03
p-Coumaric acid	10.730 ± 0.33	16.601 ± 0.15
Ferulic acid	1.512 ± 0.05	0.815 ± 0.02
Quercetin-3-glucoside	14.259 ± 0.43	15.842 ± 0.13
Quercetin-3-rhamnoside	77.868 ± 2.31	47.275 ± 1.01
Myricetin	12.451 ± 0.38	14.687 ± 0.11
Kaempferol rhamnoside	81.832 ± 2.45	47.173 ± 1.02
Quercetin	83.619 ± 2.48	93.386 ± 1.98
Kaempferol	12.307 ± 0.38	0.550 ± 0.02
Apigenin	1.669 ± 0.06	0.206 ± 0.01

## Data Availability

The raw data supporting the conclusions of this article will be made available by the authors on request.
